# Attenuation of the Type IV Pilus Retraction Motor Influences *Neisseria gonorrhoeae* Social and Infection Behavior

**DOI:** 10.1128/mBio.01994-16

**Published:** 2016-12-06

**Authors:** Alyson M. Hockenberry, Danielle M. Hutchens, Al Agellon, Magdalene So

**Affiliations:** a Department of Immunobiology, University of Arizona, Tucson, Arizona, USA; b BIO5 Institute, University of Arizona, Tucson, Arizona, USA; c School of Animal and Comparative Biomedical Sciences, University of Arizona, Tucson, Arizona, USA

## Abstract

Retraction of the type IV pilus (Tfp) mediates DNA uptake, motility, and social and infection behavior in a wide variety of prokaryotes. To date, investigations into Tfp retraction-dependent activities have used a mutant deleted of PilT, the ATPase motor protein that causes the pilus fiber to retract. Δ*pilT* cells are nontransformable, nonmotile, and cannot aggregate into microcolonies. We tested the hypothesis that these retraction-dependent activities are sensitive to the strength of PilT enzymatic activity by using the pathogen *Neisseria gonorrhoeae* as a model. We constructed an *N. gonorrhoeae* mutant with an amino acid substitution in the PilT Walker B box (a substitution of cysteine for leucine at position 201, encoded by *pilT*_L201C_). Purified PilT_L201C_ forms a native hexamer, but mutant hexamers hydrolyze ATP at half the maximal rate. *N. gonorrhoeae pilT*_L201C_ cells produce Tfp fibers, crawl at the same speed as the wild-type (wt) parent, and are equally transformable. However, the social behavior of *pilT*_L201C_ cells is intermediate between the behaviors of wt and Δ*pilT* cells. The infection behavior of *pilT*_L201C_ is also defective, due to its failure to activate the epidermal growth factor receptor (EGFR)-heparin-binding EGF-like growth factor (HB-EGF) pathway. Our study indicates that pilus retraction, *per se*, is not sufficient for *N. gonorrhoeae* microcolony formation or infectivity; rather, these activities are sensitive to the strength of PilT enzymatic activity. We discuss the implications of these findings for *Neisseria* pathogenesis in the context of mechanobiology.

## IMPORTANCE

Type IV pili are fibers expressed on the surface of many bacteria. *Neisseria gonorrhoeae* cells crawl, take up DNA, and communicate with each other and with human cells by retracting these fibers. Here, we show that an *N. gonorrhoeae* mutant expressing an enzymatically weakened type IV pilus retraction motor still crawls and takes up DNA normally. However, mutant cells exhibit abnormal social behavior, and they are less infective because they fail to activate the epidermal growth factor receptor. Our study shows that *N. gonorrhoeae* social and infection behaviors are sensitive to the strength of the retraction motor enzyme.

## INTRODUCTION

Type IV pili (Tfp) are produced by many prokaryotes, including members of the *Archaea* (1). The organelle promotes attachment, motility, and DNA uptake (horizontal gene transfer) (2–8). Tfp also plays an important role in the social behavior of bacterial cells, facilitating biofilm formation and host cell signaling (3, 9–13). These activities require the physical retraction of the Tfp fiber.

The Tfp retraction motor is composed of six subunits of the ATPase associated with various activities (AAA) protein, PilT (1). ATP hydrolysis by the subunits causes conformational changes in the hexamer that are transduced into mechanical energy (14, 15). In the case of the prototypical *Pseudomonas aeruginosa* motor, rounds of ATP binding, hydrolysis, and release alter the conformation of its subunits (16–18), which, by a poorly understood mechanism, causes the pilus fiber to retract.

Tfp retraction and its biological consequences are well studied in *Neisseria* (2, 10–12, 19). In the human pathogen *Neisseria gonorrhoeae*, the PilT ATPase serves as the pilus retraction motor (2, 16, 19). An *N. gonorrhoeae* mutant deleted of PilT (Δ*pilT*) is nonmotile and nontransformable (2, 19), and mutant cells cannot form microcolonies (biofilm precursors) (13, 20).

Tfp retraction also influences *N. gonorrhoeae* infection behavior (10–12, 21–23). Cycles of pilus assembly, substrate tethering, and retraction cause a massive reorganization of the infected host cell cortex and activate cytoprotective signaling pathways that skew infection outcomes in favor of host and pathogen (11, 12, 22, 24, 25). The retraction of the pilus fiber exerts a significant amount of force (19, 25–28). Many host cell responses to *N. gonorrhoeae* infection are known to be caused by this mechanical stimulation (12), but whether these responses are sensitive to variations in the pilus retraction force is unknown.

During infection, pathogenic *Neisseria* activates the epidermal growth factor receptor (EGFR) pathway (22, 29–31), and disrupting this pathway reduces the number of viable *N. gonorrhoeae* cells recovered from within the infected cell (29). Whether pilus retraction activates EGFR is unknown.

To date, studies of Tfp retraction-dependent events have used a mutant with a deletion mutation of *pilT* (Δ*pilT*). This approach, though useful, can only identify all or none phenotypes. Here, we took a different approach. We constructed an *N. gonorrhoeae* mutant, *pilT*_L201C_ (encoding a PilT mutant in which cysteine replaces leucine at position 201), that expresses a Tfp retraction motor with half-maximal ATPase activity and characterized the biological activities known to require Tfp retraction. We report that *pilT*_L201C_ retains the ability to retract Tfp. It crawls at the same speed and takes up DNA at the same frequency as the wild-type (wt) parent. It also attaches to epithelial cells equally well. However, the social behavior of *pilT*_L201C_ cells is intermediate between those of wt and Δ*pilT* cells. The infection behavior of *pilT*_L201C_ is also defective, due to its inability to activate the EGFR-heparin-binding EGF-like growth factor (HB-EGF) pathway. Δ*pilT* is also defective in this regard. These findings show that EGFR-HB-EGF activation requires Tfp retraction, that there is a threshold for activating this pathway, and that a PilT motor with reduced ATPase activity is insufficient to overcome this threshold. Overall, our study indicates that some but not all Tfp retraction-dependent activities are sensitive to the strength of PilT enzymatic activity.

## RESULTS

### An amino acid substitution in the PilT Walker B domain in *N. gonorrhoeae* PilT attenuates its ATP hydrolysis rate.

**FIG 1:**
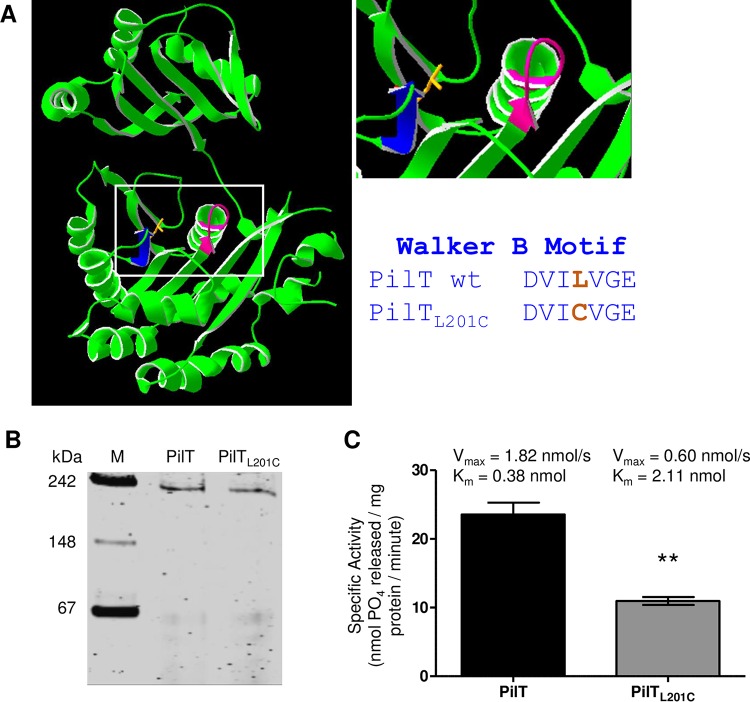
PilT_L201C_ forms a hexamer but has reduced ability to bind and hydrolyze ATP. (A) Predicted structure of the *N. gonorrhoeae* PilT monomer (left), close-up view of its ATP binding site (top right), and residues of the Walker B domain of wt PilT and PilT_L201C_ (bottom right). Walker A domain (magenta); Walker B domain (blue); L201 side chain (orange). (B) Migration of purified PilT and PilT_L201C_ in a nondenaturing 6.5% acrylamide gel stained with Coomassie blue. Predicted molecular mass of the PilT hexamer is 222 kDa. (C) ATPase activity, *V*_max_, and *K*_m_ values of PilT and PilT_L201C_ hexamers. Values are the average results from 3 independent experiments, each performed in triplicate. **, *P* < 0.01, Student’s two-tailed unpaired *t* test.

Sequence, biochemical, and structural analyses of PilT orthologues strongly suggest that *N. gonorrhoeae* PilT is a member of the AAA protein family. Freeze-etch microscopy shows that it is disc-shaped, with six subunits (32). Sequence-based structural predictions reveal extensive homology between *N. gonorrhoeae* PilT and its *Pseudomonas aeruginosa* orthologue (Fig. 1A) (16, 17). To confirm that *N. gonorrhoeae* PilT is an ATPase, we cloned, overexpressed, and purified the protein from *Escherichia coli*. Recombinant PilT migrated as a hexamer in a nondenaturing polyacrylamide gel (Fig. 1B). The hexamer hydrolyzed ATP at the rate of 24.9 nmol PO_4_/mg PilT/minute at 37°C (Fig. 1C), similar to the ATPase activities of motor proteins from other type IV pilus (Tfp) and type II secretion systems (33, 34).

In AAA proteins, the Walker A and B boxes work in conjunction to catalyze ATP hydrolysis (14). Mutations in the Walker A and B domains affect the rate at which they hydrolyze ATP (35, 36). We mutated a single amino acid in the PilT Walker B domain, exchanging the leucine at position 201 for a cysteine (L201C). We chose cysteine because, with this substitution, the PilT Walker B domain becomes identical to the Walker B domain of PilU, a closely related AAA protein implicated as taking part in retracting Tfp (Fig. 1A). This substitution is less likely to inadvertently alter the conformation of the motor. The *pilT*_L201C_ allele does not exist in the 820 *Neisseria pilT* alleles listed in the BIGSDb database (37). Modeling of PilT_L201C_ showed minimal changes in protein structure compared to that of wt PilT (see Fig. S1 in the supplemental material).

Purified PilT_L201C_ assembled into hexamers that migrated at the molecular mass of wt PilT in nondenaturing gels (Fig. 1B). However, the PilT_L201C_ hexamer hydrolyzed ATP at a rate of 13.2 nmol PO_4_/mg PilT_L201C_/minute, which is approximately 50% of the specific activity of wt PilT (Fig. 1C). The mutant hexamer had a higher *K*_*m*_ and lower *V*_max_ than wt PilT (Fig. 1C), indicating it has a lower affinity for ATP and a reduced ability to hydrolyze the substrate. We conclude that the L201C mutation in the Walker B domain attenuates the ATPase activity of PilT but does not affect its native conformation.

### *N. gonorrhoeae pilT*_L201C_ retains the ability to retract pili.

**FIG 2:**
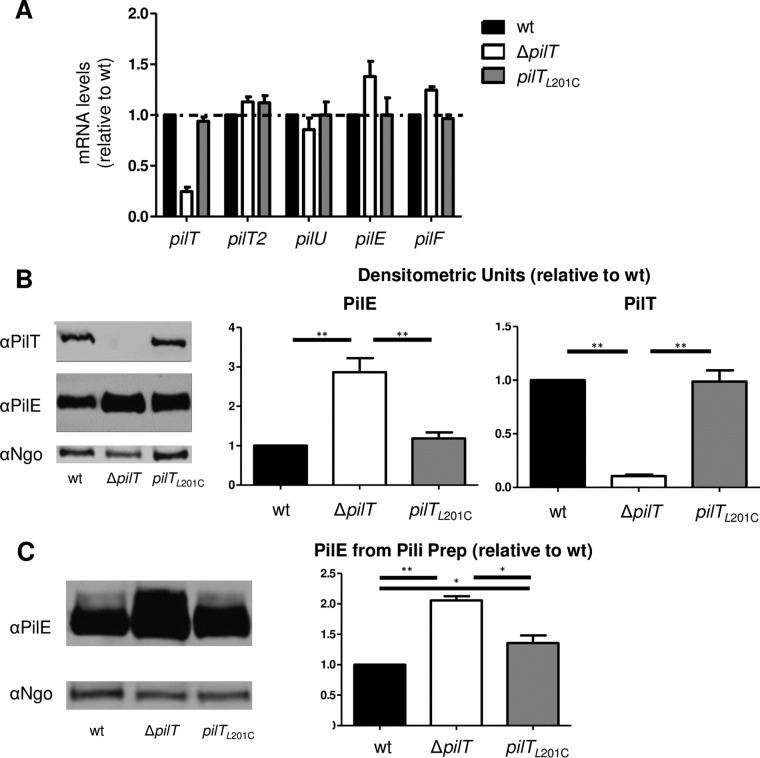
*N. gonorrhoeae pilT*_L201C_ produces more pili but not more *pilE* mRNA or protein than the wt. (A) mRNA levels in log-phase bacteria were determined by real-time PCR using primers specific for the 16S rRNA gene, *pilT*, *pilT2*, *pilU*, *pilE*, and *pilF* (see Table S2 in the supplemental material). mRNA levels were normalized to the value for the 16S rRNA gene, and the values are expressed relative to those of the wt. Values are the average results from 3 independent experiments. (B) (Left) A representative Western blot of whole-cell lysates from log-phase bacteria probed with anti-PilT antibody, anti-PilE antibody, and a rabbit anti-*N. gonorrhoeae* antibody (αNgo) raised against whole *N. gonorrhoeae* cells. (Right) Levels of PilE and PilT were determined from 4 independent experiments. PilE and PilT densitometry signals were normalized to that of an invariant *N. gonorrhoeae* protein in the same lane. **, *P* < 0.01, Student’s two-tailed unpaired *t* test. (C) (Left) A representative Western blot of crude pilus preparation from PilT_L201C_ cells using anti-PilE antibody and anti-*N. gonorrhoeae* antibody. (Right) Levels of extracellular PilE were determined from 4 independent experiments. The PilE signal was normalized to that of an invariant *N. gonorrhoeae* protein in the same lane. *, *P* < 0.05, and **, *P* < 0.01, Student’s two-tailed unpaired *t* test.

We replaced the wt copy of *pilT* in *N. gonorrhoeae* with the *pilT*_L201C_ allele. *N. gonorrhoeae pilT*_L201C_ grew as well as the wt parent in liquid (data not shown). *N. gonorrhoeae* Δ*pilT* is known to be hyperpiliated and to produce significantly higher levels of PilE and *pilE* mRNA than the wt (2, 38). We found that *N. gonorrhoeae pilT*_L201C_ transcribed the Tfp-associated genes *pilE*, *pilT*, *pilT2*, *pilU*, and *pilF* at wt levels (Fig. 2A). In our assays, *N. gonorrhoeae* Δ*pilT* consistently produced more *pilE* mRNA than the wt, but this difference was not statistically significant. Western blotting for PilE in whole-cell lysates and pilus preparations showed that *N. gonorrhoeae* Δ*pilT* produced more total PilE and more pili than the wt (Fig. 2B and C), confirming earlier reports. *N. gonorrhoeae pilT*_L201C_ produced wt levels of PilT and total PilE (Fig. 2B), as might be predicted from the mRNA data described above. However, it produced pili at a level intermediate between the levels produced by wt and Δ*pilT N. gonorrhoeae* (Fig. 2C). This is consistent with the intermediate ATPase activity of PilT_L201C_.

**FIG 3:**
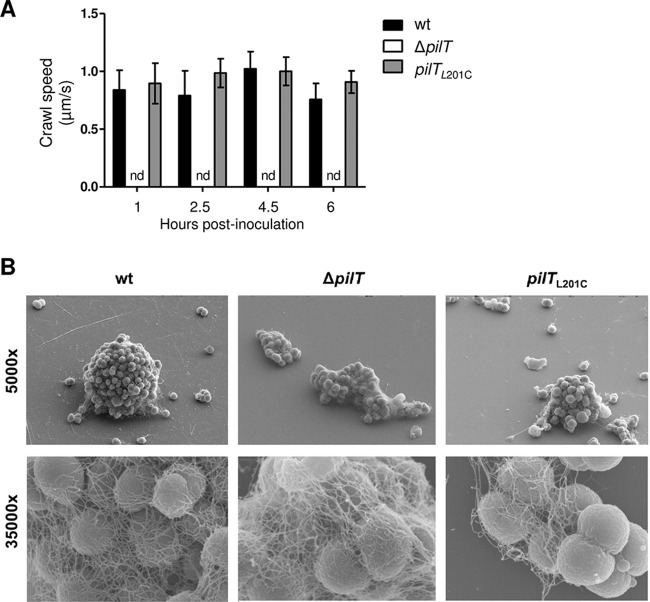
*N. gonorrhoeae pilT*_L201C_ is motile but forms aberrant microcolonies. (A) Crawl speeds of 50 individual cells from each strain were tracked over 30 s at various time points postinoculation. Values are the average results from 3 independent experiments. nd, not detected. (B) Scanning electron micrographs of *N. gonorrhoeae* wt, Δ*pilT*, and *pilT*_L201C_ grown on a glass slide for 4 h. Magnification, ×5,000 (top); ×35,000 (bottom).

We focused our attention on the biological impact of the L201C mutation. (A separate study is under way to measure the physical properties of pilus retraction in *N. gonorrhoeae pilT*_L201C_.) We determined whether *pilT*_L201C_ retracts its Tfp fibers by using DNA transformation and twitching motility as readouts. The mutant is transformed by *N. gonorrhoeae* chromosomal DNA at approximately the same frequency as the wt (Table 1). As expected, the transformation frequency of *N. gonorrhoeae* Δ*pilT*, the negative control, was below the limit of detection. *pilT*_L201C_ cells crawled at ~1 µm/s, the speed reported for cells of the wt parent (Fig. 3A). The Δ*pilT* control was nonmotile. Thus, reducing the enzymatic activity of the PilT motor did not affect Tfp retraction, *per se*, or the retraction-dependent activities of DNA transformation and twitching motility.

**TABLE 1:** Transformation efficiency of *N. gonorrhoeae* *pilT*_L201C_

Strain	Transformation frequency^a^	
	− DNA	+ DNA
Wild type	<4.97 × 10^−6^^b^	2.65 × 10^−4^ ± 1.35 × 10^−4^
Δ*pilT*	<9.40 × 10^−6^^b^	<6.44 × 10^−6^^b^
*pilT*_L201C_	<4.97 × 10^−6^^b^	1.69 × 10^−4^ ± 0.65 × 10^−4^

^a^ Transformation frequency = Rif^r^ CFU/total CFU/µg Rif^r^ genomic DNA. Values are the average results from 4 independent experiments ± standard errors of the means. Student’s two-tailed *t* test.

^b^ Limit of detection.

Microcolonies are biofilm precursors. *N. gonorrhoeae* cells crawl together to form microcolonies on synthetic substrates and epithelial cells. Microcolonies move, and they crawl toward each other to fuse into larger, motile structures. Bacteria within two or more fusing microcolonies rearrange their positions relative to each other, eventually producing a single tall, densely packed sphere. Δ*pilT* cells do not form microcolonies, as this trait requires crawling, but many are observed in shapeless clusters (10, 11, 22). *N. gonorrhoeae pilT*_L201C_ had an intermediate microcolony formation phenotype on abiotic surfaces: *pilT*_L201C_ cells formed microcolonies, as predicted from their motile state, but these structures were shorter, smaller, and less spherical (Fig. 3B). Differences in the phenotypes of wt, *pilT*_L201C_, and Δ*pilT* microcolonies were also observed on human epithelial cells (see Fig. S2 in the supplemental material).

The phenotype of an *N. gonorrhoeae pilT*_L201C_ biofilm is also intermediate between those of the wt and Δ*pilT* strains, as determined by crystal violet staining of static cultures after 4, 8, and 24 h of growth (see Fig. S2A in the supplemental material). *pilT*_L201C_ biofilms retained significantly more crystal violet than wt biofilms at all time points. Δ*pilT* biofilms also retained more dye than wt biofilms, this difference being very noticeable after 8 h of static growth. Finally, wt, *pilT*_L201C_ and Δ*pilT* biofilms have distinctly different morphologies (see Fig. S3B). These results further illustrate the differences in the social/community behaviors of *pilT*_L201C_ and Δ*pilT* cells and emphasize the importance of PilT ATP hydrolysis in this process.

### The invasiveness defect of *pilT*_L201C_ is due to its inability to activate the EGFR-HB-EGF pathway.

**FIG 4:**
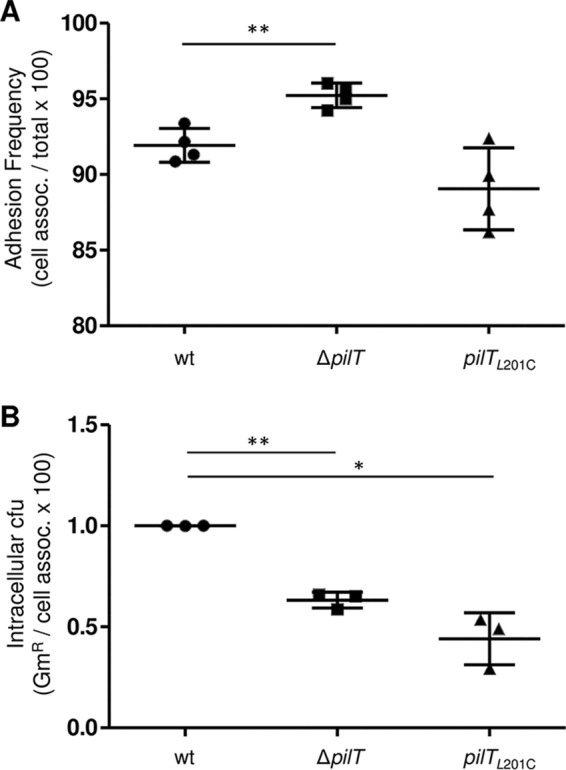
Fewer viable *N. gonorrhoeae pilT*_L201C_ cells than wt *N. gonorrhoeae* cells are found inside epithelial cells. ME180 cells were infected with *N. gonorrhoeae* wt, Δ*pilT*, and *pilT*_L201C_ for 4 h, and adhesion (A) and numbers of intracellular bacteria (B) were determined. The numbers of gentamicin-protected *N. gonorrhoeae* Δ*pilT* and *pilT*_L201C_ CFU were normalized to the number of gentamicin-protected wt *N. gonorrhoeae* CFU. Values are the average results from 3 or more independent experiments. *, *P* < 0.001, and **, *P* < 0.01, Student’s two-tailed paired *t* test.

Wild-type and Δ*pilT* mutants of pathogenic *Neisseria* strains attach to epithelial cells equally well, indicating that Tfp retraction is not crucial for the adherence phase of infection (11, 20, 39). However, in gentamicin (Gm) protection assays, fewer viable Δ*pilT* cells are recovered from within host cells (11), indicating that Tfp retraction influences bacterial infectivity. We determined whether *N. gonorrhoeae pilT*_L201C_ is defective in infection. *pilT*_L201C_ cells adhered to ME180 human epithelial cells as well as the wt parent cells (Fig. 4A). As reported previously, Δ*pilT* cells adhered slightly better than wt cells due to their higher levels of piliation (11, 20, 39). Gm protection assays yielded roughly equal numbers of intracellular Δ*pilT* and *pilT*_L201C_ CFU, these values being approximately half the wt CFU count (Fig. 4B). Thus, a mutant expressing an attenuated PilT motor is as infectious as a Δ*pilT* mutant.

**FIG 5:**
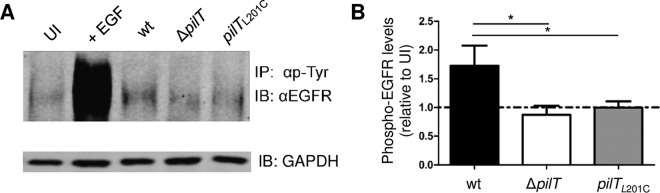
*N. gonorrhoeae* Δ*pilT* and *pilT*_L201C_ cells have reduced ability to stimulate EGFR phosphorylation. (A) A typical Western blot of phosphorylated EGFR levels in *N. gonorrhoeae* wt-, Δ*pilT*-, and *pilT*_L201C_-infected ME180 cells. UI, uninfected control; IP, immunoprecipitation; IB, immunoblotting. (B) Phospho-EGFR signals averaged from four Western blots. Phospho-EGFR density was normalized to the signal for the internal GAPDH control and expressed relative to the value from the uninfected control.

A study showed that *N. gonorrhoeae* invasion of epithelial cells requires activation (phosphorylation) of the epidermal growth factor receptor (EGFR) (29, 40). The role of Tfp retraction in this process was not examined. We tested the ability of Δ*pilT* and *pilT*_L201C_ *N. gonorrhoeae* to trigger EGFR phosphorylation. Whole-cell lysates of ME180 cells infected for 4 h with wt, Δ*pilT*, and *pilT*_201C_ strains were immunoprecipitated with an anti-phosphotyrosine antibody, and the precipitates were immunoblotted for EGFR. Uninfected cells and EGF-stimulated uninfected cells served as the negative and positive controls, respectively. Compared to wt-infected cells, Δ*pilT* and *pilT*_L201C_ *N. gonorrhoeae*-infected cells had lower and comparable levels of phospho-EGFR (Fig. 5A and B).

**FIG 6:**
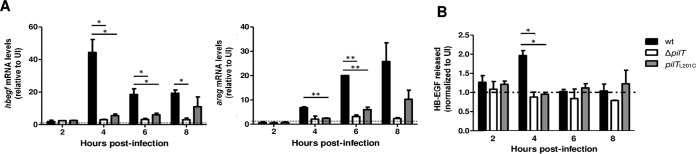
*N. gonorrhoeae ΔpilT* and *pilT*_L201C_ cells fail to induce transcription of EGFR ligands and shedding of HB-EGF. (A) mRNA levels of *hbegf* (left) and *areg* (right) in *N. gonorrhoeae* wt-, Δ*pilT*- and *pilT*_L201C_-infected cultures were determined by real-time PCR; the values were normalized to the value for the GAPDH internal control and expressed relative to the value for the uninfected control (UI). Values are the average results from 3 independent experiments. *, *P* < 0.05, and **, *P* < 0.01, Student’s two-tailed unpaired *t* test. (B) Results of Western dot blot of supernatants from infected cultures for soluble HB-EGF. Signals were normalized to the value for uninfected-cell supernatant. Values are the average results from 3 independent experiments. *, *P* < 0.05, Student’s two-tailed unpaired *t* test.

Metalloproteinases cleave EGFR ligands from the plasma membrane, releasing their soluble ectodomains to bind and activate EGFR (41, 42). Upon binding ligand, EGFR forms a homodimer or a heterodimer with Her2/Erb2; this in turn stimulates *trans*-phosphorylation of the paired cytosolic tails (42). *N. gonorrhoeae* upregulates the transcription of two EGFR ligands, heparin-binding epidermal growth factor (*hbegf*) and amphiregulin (*areg*) (12, 29). We determined whether Tfp retraction upregulates and induces the shedding of HB-EGF and amphiregulin. Using real-time PCR, *hbegf* and *areg* mRNA were quantitated in ME180 cells infected with wt, Δ*pilT*, or *pilT*_L201C_ *N. gonorrhoeae* for 2, 4, 6, or 8 h. The wt strain upregulated both transcripts. *hbegf* mRNA peaked at 4 h postinfection (hpi) and decreased to lower but still significant levels thereafter (Fig. 6). *areg* mRNA increased gradually, reaching a high level at the last time point. Δ*pilT* and *pilT*_L201C_ *N. gonorrhoeae* failed to upregulate *hbegf* and *areg* compared to the results for wt *N. gonorrhoeae*. The *pilT*_L201C_ strain appeared to upregulate *hbegf* and *areg* more strongly than the Δ*pilT* strain, though this increase was not statistically significant at any of the time points tested. The wt strain did not affect the transcription of epidermal growth factor (*egf*), as reported previously (29), nor did the Δ*pilT* or *pilT*_L201C_ strain (data not shown).

To determine whether Tfp retraction stimulates HB-EGF ectodomain shedding, we quantitated its levels in supernatants of ME180 cells infected with wt, Δ*pilT*, or *pilT*_L201C_ *N. gonorrhoeae*. Supernatants from wt-infected cultures had higher levels of soluble HB-EGF at 4 hpi (Fig. 6B), coincident with the time at which *hbegf* mRNA levels peaked (Fig. 6A). In contrast, supernatants from Δ*pilT*- and *pilT*_L201C_ strain-infected cultures had no detectable HB-EGF. Together, these results indicate that upregulation of EGFR ligands HB-EGF and amphiregulin and the shedding of HB-EGF require a PilT motor with higher enzymatic activity than PilT_L201C_.

**FIG 7:**
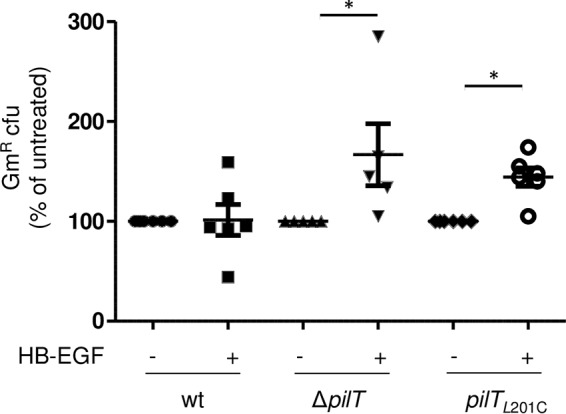
Addition of exogenous HB-EGF increases the numbers of viable intracellular *N. gonorrhoeae* Δ*pilT* and *pilT*_L201C_ cells. CFU counts of gentamicin-protected cells from cultures infected for 4 h with wt, Δ*pilT*, or *pilT*_L201C_ cells and treated with HB-EGF at 3 h postinfection. Values were normalized to CFU counts from mock-treated, similarly infected samples and are expressed as percentages of the wt value. Values are the average results from 6 independent experiments. *, *P* < 0.05, and **, *P* < 0.01, Student’s two-tailed paired *t* test.

Finally, we determined whether adding HB-EGF to infected cultures could rescue the infectivity defect of these mutants. Soluble HB-EGF was added to wt-, Δ*pilT*- and *pilT*_L201C_ strain-infected cultures at 3.5 hpi at a concentration (6 ng/ml) equivalent to the peak detected in wt-infected supernatants (Fig. 6B). Thirty minutes later, adhered and intracellular bacteria were quantitated. The presence of HB-EGF did not alter bacterial adherence for any of the strains (data not shown) or affect the amount of wt intracellular CFU recovered. However, it increased intracellular Δ*pilT* and *pilT*_L201C_ CFU by approximately 50% each (Fig. 7). Thus, the invasiveness defect of both Δ*pilT* and *pilT*_L201C_ *N. gonorrhoeae* can be partially rescued by adding HB-EGF *in trans*.

Taken together, these results show that Tfp retraction plays an important role in the transcriptional upregulation and proteinase processing of HB-EGF, which, in turn, activates EGFR and promotes *N. gonorrhoeae* infectivity. These data also show that *N. gonorrhoeae* expressing an attenuated PilT motor is as incapable of stimulating this pathway as a Δ*pilT* mutant.

## DISCUSSION

To carry out many of its functions, the type IV pilus has to retract (1). To date, Tfp retraction studies have used mutants deleted of the *pilT* gene. Whether Tfp retraction-dependent activities are sensitive to the relative enzymatic activity of the PilT motor has not been tested. To examine this issue, we constructed an *N. gonorrhoeae* mutant that expresses a PilT motor with reduced enzymatic activity and examined its Tfp retraction-related behaviors (see Table S1 in the supplemental material). This mutant protein, PilT_L201C_, has a single amino acid substitution in the Walker B domain. The L201C mutation does not affect the ability of PilT_L201C_ to form native hexamers; however, it lowers the ATP binding affinity of the hexamer and decreases its ATP hydrolysis rate by 50%.

*N. gonorrhoeae pilT*_L201C_ has retained the ability to retract Tfp, as judged by the wt transformation frequency and crawl speed. It differs from the wt and Δ*pilT* strains in its social behavior (Fig. 3). Unlike wt cells, which aggregate into spherical microcolonies, and Δ*pilT* cells, many of which are found in amorphous aggregates, *pilT*_L201C_ cells form misshapen microcolonies, suggesting a failure of *pilT*_L201C_ cells to sense/signal each other. As microcolonies are biofilm precursors, these findings have implications for *N. gonorrhoeae* infectivity.

It is unclear how PilT_L201C_ affects *N. gonorrhoeae* intercellular communication. One possible explanation is that it is indirectly involved in interbacterial mechanical sensing and signaling. The *Pseudomonas aeruginosa* Tfp biogenesis protein PilY1 is a mechanosensor that regulates bacterial gene expression and behavior in response to attachment (43). In *N. gonorrhoeae*, the Tfp-associated proteins PilC1 and PilC2 are PilY1 orthologues. The ability of PilC to sense/respond to mechanical stress has yet to be tested, but both contain a putative von Willebrand factor A domain that is posited to be involved in *P. aeruginosa* mechanosensing (43, 44). The PilC protein(s) may operate on a similar principle, allowing an *N. gonorrhoeae* cell to sense the resistance from neighboring cells tethered to its retracting Tfp and coordinate community and infection behavior through transcriptional reprogramming. In support of this hypothesis, the Δ*pilT* mutant displays a different transcriptional profile than its wt parent (38).

*N. gonorrhoeae* *pilT*_L201C_ and Δ*pilT* cells attach to epithelial cells, but both are less invasive than the wt, and to the same degree (Fig. 4). *pilT*_L201C_ cells, which can retract Tfp, and Δ*pilT* cells, which cannot retract Tfp, behave similarly in infection assays (Fig. 4 to 7). This strongly suggests that not all Tfp retraction events are sufficient for *N. gonorrhoeae* infectivity.

The infection defect of *pilT*_L201C_ and Δ*pilT* cells is due to their inability to signal through the EGFR pathway (Fig. 5, 6, and 7). Both mutants fail to activate EGFR, in part because they cannot upregulate two of its ligands, HB-EGF and amphiregulin, or stimulate the release of the biologically active HB-EGF ectodomain. Both the transcriptional upregulation and the release of active HB-EGF are stimulated by mechanical stress (45–47). The mechanical forces generated by pilus retraction in *pilT*_L201C_ cells remain to be defined, but our findings are consistent with the mechanosensitive nature of EGFR activation (45–47). Our data suggest that there is a threshold for EGFR-HB-EGF activation and that the PilT_L201C_ motor, with its reduced ATPase activity, cannot provide the required mechanical stimulus. The ability of *pilT*_L201C_ *N. gonorrhoeae* to stimulate other Tfp retraction-induced pathways within the epithelial cell is under investigation.

The activation of the EGFR-HB-EGF pathway has been shown to promote *N. gonorrhoeae* invasiveness (29, 30). In addition to corroborating this observation, we showed that the activation of this pathway requires Tfp retraction. Whether the invasiveness promoted by the EGFR-HB-EGF pathway is due to accelerated bacterial entry, increased intracellular survival, or both is unclear. Many residues in the EGFR tail can be phosphorylated, and the phosphorylation pattern determines which downstream signaling cascades are activated (48). The full extent of the EGFR signaling programs induced by Tfp retraction and their consequences for infection remain to be investigated.

Our study emphasizes the need to examine additional PilT mutants with various degrees of enzymatic activity and to define the relationship between PilT enzymatic activity and Tfp retraction dynamics. That the retractile Tfp fibers of *pilT*_L201C_ cells cannot stimulate the mechanosensitive EGFR pathway implies that Tfp retraction dynamics (force, speed, and/or frequency) modulate the host response to *N. gonorrhoeae* infection. In this context, it is also interesting to note that environmental cues influence Tfp retraction speed and force (25–27, 49, 50). Oxygen influences the retraction speed of a Tfp fiber (26). Tfp fibers form bundles under certain conditions, and these bundles retract with dramatically higher force than single fibers (25). The bodily niches for *Neisseria* vary in oxygen tension, type and level of nutrients, and temperature. Thus, Tfp retraction dynamics and, by implication, bacterial social and infection behavior might differ in the various niches. The quality and quantity of Tfp retraction events could determine which host signaling pathways are activated and/or modulate pathway signal strength. This in turn could determine whether an *N. gonorrhoeae* infection becomes asymptomatic or inflammatory.

## MATERIALS AND METHODS

### Bacterial strains, growth conditions, and infection studies.

*Neisseria gonorrhoeae* strains MS11 and MS11 Δ*pilT* (38) were used throughout this study. All strains were grown on gonococcal broth (GCB) agar plates supplemented with V-C-N (vancomycin, colistin, and nystatin) inhibitor (BBL; BD) or in liquid GCB containing Kellogg’s supplements I and II at 37°C with 5% CO_2_. *Escherichia coli* strains DH5α and BL21 were grown in Luria broth at 37°C.

The human endocervical epithelial cell line ME180 (ATCC no. HTB-33, 9 to 18 passages) was used throughout this study. Cells were maintained in Falcon tissue culture dishes in RPMI 1640 plus glutamine (Gibco) supplemented with 10% fetal bovine serum (FBS; Sigma). For all experiments, cells were seeded into wells 2 days prior to the day of the experiment. After 24 h, the epithelial cells were serum starved by culturing in serum-free RPMI 1640 plus glutamine. After 16 h of serum starvation, serum-free medium was removed and replaced with RPMI 1640 plus glutamine supplemented with 10% FBS, and the cells infected with bacteria. Details regarding seeding densities and confluence at the time of infection are listed with the respective experimental procedures below.

### PilT structure prediction.

Structural predictions for *N. gonorrhoeae* PilT and PilT_L201C_ proteins were performed using the Phyre server (http://www.sbg.bio.ic.ac.uk/phyre/) and based on the structure of *Pseudomonas aeruginosa* PilT (PDB model 3JVU). The resultant files were examined using Swiss PDBViewer (version 4.1).

### Cloning and site-directed mutagenesis of *pilT*.

For protein overexpression, the *pilT* gene of *N. gonorrhoeae* was amplified using Phusion polymerase (New England Biolabs) and primers as indicated in Table S2 in the supplemental material. This PCR product was digested with SacI and BamHI (New England Biolabs) and ligated into similarly digested pET28a (Novagen) using T4 ligase (New England Biolabs), which leaves the N-terminal His_6_ tag and linker sequence intact. Overlap extension mutagenesis was employed to introduce the L201C mutation into the *pilT* PCR product (51). This product was cloned into pET28a as described above. All constructs were verified by DNA sequencing (Elim Biopharmaceuticals).

### Overexpression and purification of PilT.

An overnight culture of *E. coli* BL21 bearing the plasmid pET28a-*pilT* or pET28a-*pilT*_L201C_ was inoculated into 100 ml LB supplemented with kanamycin (50 mg/liter) and chloramphenicol (30 mg/liter) to an optical density at 600 nm (OD_600_) of 0.1. The cultures were incubated at 37°C with shaking for 3 h. IPTG was added to the cultures to a final concentration of 1 mM, and the cultures were incubated for an additional 3 h. The cultures were centrifuged, and cell pellets were stored at −20°C for at least 16 h. Cell pellets were thawed and resuspended in Tris (100 mM, pH 8.5) containing glycerol (10%), KCl (300 mM), lysozyme (100 mg/liter), and DNase (100 mg/liter). This suspension was incubated at room temperature, with 3 1-min vortexing sessions every 10 min. The sample was then centrifuged, the supernatant was applied to Ni-nitrilotriacetic acid (NTA) resin (Roche), and the column was incubated with rotation overnight at 4°C. The PilT proteins were purified using a spin column method (Qiagen). The flowthrough was collected, and the column was washed with 10 resin volumes of wash buffer (Tris [150 mM, pH 8.5], KCl [300 mM], glycerol [10%], and imidazole [20 mM]). The bound proteins were then eluted from the column with elution buffer (Tris [150 mM, pH 8.5], KCl [300 mM], glycerol [10%], and imidazole [200 mM]). Fraction samples were separated on a 10% acrylamide SDS-PAGE gel to confirm purity. Purified PilT was concentrated and spin dialyzed using 3,000 molecular-weight cutoff (3K MWCO) columns (Eppendorf) into Tris (100 mM, pH 8.5), KCl (300 mM), and glycerol (10%). Purified proteins were separated on a 6.5% acrylamide nondenaturing gel to determine whether they formed native hexamers. Protein concentration was determined by the bicinchoninic acid (BCA) assay (Pierce) according to the manufacturer’s instructions.

### ATPase assays.

The ATP hydrolysis rates of purified PilT and PilT_L201C_ were tested using BioMol green reagent (Enzo Biosciences) according to the manufacturer’s recommendations. At time zero, 5 mM ATP was added to 100 ng protein in ATPase assay buffer (Tris [150 mM, pH 8.5], NaCl [150 mM], and MgCl_2_ [5 mM]) preequilibrated to 37°C. The phosphate levels were measured at 2, 5, 10, 15, and 20 min after the addition of ATP. The values given are the average results of triplicate assays using 3 separate experiments from 3 separate protein preparations (27 assays total). *K*_*m*_ and *V*_max_ values were determined by testing each protein with a variable amount of ATP in the reaction mixture and measuring the phosphate levels at 10 min after the addition of ATP.

### Construction of the *N. gonorrhoeae pilT*_L201C_ mutant.

The *pilT*_L201C_ allele was cloned into the endogenous *pilT* locus using an allelic replacement approach. The promoter and kanamycin resistance gene were amplified from pMR68 using primers AH107F and -R. This product was then amplified using primers AH107F and AH108 to add the 3′ untranslated region (UTR) of *pilU* and inserted into the BamHI/HincII site of pUC19 to create pUC19-Kan. The *N. gonorrhoeae pilTU* operon was amplified using primers 106F and -R, and then the L201C mutation was introduced into this product using overlap extension PCR (51). The *pilTU* operon was then inserted into pUC19-Kan at the SmaI/BamHI site to create pUC19-*pilTU*Kan (see Fig. S2 in the supplemental material). *N. gonorrhoeae* MS11 was transformed with pUC19-*pilTU*Kan and selected for on GCB agar plates supplemented with kanamycin (50 mg/liter). The *pilT* locus of kanamycin-resistant transformants was sequenced to confirm the mutation of L201 (8/10 Km^r^ clones had the L201C mutation). The genomic DNA from one clone was backcrossed into wt *N. gonorrhoeae* MS11. The *pilT*, *pilU, pilT2*, and *pilE* loci from backcrossed kanamycin-resistant clones were sequenced to confirm the absence of mutations.

### Quantitating transcript and protein levels in *N. gonorrhoeae*.

Bacterial strains were inoculated into 25 ml of GCB containing Kellogg’s supplements I and II at an OD_600_ of 0.01 and incubated at 37°C for 4 h with shaking. The cultures were split into two 12.5-ml portions and centrifuged to harvest the bacteria. Pellets were resuspended in either 1 ml Trizol (Invitrogen) for mRNA analysis or 200 µl radioimmunoprecipitation assay (RIPA) buffer (sodium phosphate [10 mM, pH 7.2], NaCl [150 mM], EDTA [5 mM], NaF [50 mM], SDS [0.1%], deoxycholate [1%], Triton X-100 [1%]) containing protease inhibitors for protein analysis.

Bacterial mRNA extraction and cDNA synthesis were performed as previously described (52). Real-time PCR mixtures contained cDNA template, gene-specific primers (see Table S2 in the supplemental material), and SYBR green PCR master mix (Invitrogen) according to the manufacturer’s instructions. Samples were analyzed on an Applied Biosystems Prism 7300 real-time PCR system. mRNA transcript levels were determined by normalization to 16S RNA levels.

PilT and PilE protein levels were determined by Western blotting of whole-cell lysate. Whole-cell lysates were boiled, and 20 µl of the lysate was separated by SDS-PAGE (15% acrylamide). The separated proteins were transferred to a nitrocellulose membrane (0.45 µm; GE Healthcare Biosciences) using the Trans-Blot SD semidry transfer cell (Life Technologies, Inc.). The membrane was blocked in Tris-buffered saline (TBS) containing 0.1% Tween 20 (TBST) and non-fat dry milk (5% wt/vol) for 1 h at room temperature and then probed with primary antibodies anti-PilE antibody (SM1) and anti-PilT antibody for 1 h at room temperature. The membrane was washed and probed with anti-rabbit and anti-mouse secondary antibodies (LICOR) diluted in 5% milk–TBST for 1 h at room temperature. The blots were imaged on the LICOR Odyssey infrared imaging system and analyzed by densitometry using ImageJ. The blots were stripped and probed with anti-*N. gonorrhoeae* antibody (an antibody raised against total *N. gonorrhoeae*), which provided internal signals as a loading control. PilT and PilE signals were normalized to the signal for an invariant *N. gonorrhoeae* protein in the same lane.

### Crude pilin preparations.

*N. gonorrhoeae* lawns were scraped from a plate and resuspended in ethanolamine (150 mM, pH 10.5), and the suspensions were vortexed for 1 min. An amount of 1 × 10^7^ bacteria was pelleted by centrifugation at 14,000 rpm for 5 min. The supernatant was collected, and to it was added ammonium sulfate (10% [wt/vol] final concentration) to precipitate the pili. Samples were incubated at room temperature for 1 h with rocking. Purified pili were pelleted by centrifugation at 14,000 rpm for 15 min. The supernatant was discarded, and the pellet was resuspended in 50 µl of ethanolamine (150 mM, pH 10.5). The samples were immunoblotted as outlined above using anti-PilE antibody (SM1) and anti-*N. gonorrhoeae* antibody for an internal loading control. The PilE signal was normalized to the signal for an invariant *N. gonorrhoeae* protein in the same lane.

### DNA transformation.

DNA transformation assays were performed as previously described (53). Briefly, 1 µg of *N. gonorrhoeae* MS11 Rif^r^ genomic DNA was added to 1 × 10^7^ bacteria in GCB containing MgSO_4_ (5 mM) and incubated for 20 min at 37°C. The cells were transferred to 900 µl prewarmed GCB with Kellogg’s supplements I and II and incubated for 2 h at 37°C at 5% CO_2_. The cells were harvested and plated on either GCB or GCB-plus-rifampicin (50 µg/liter) plates. The transformation frequency was calculated as the number of Rif^r^ CFU/total CFU/µg Rif^r^ genomic DNA. The values from 3 independent experiments were averaged.

### Motility assays.

A bacterial suspension was adjusted to an OD_600_ of 0.05 and then diluted 1:10 with GCB with Kellogg’s supplements and seeded onto a glass coverslip. The cultures were incubated at 37°C, 5% CO_2_ for 1 h to allow bacteria to settle and then measured for motility as described previously (27). Fifty individual bacteria were analyzed, each for 30 s per time point. NIS-Elements software (Nikon) was used to quantify the crawl speeds of individual bacteria.

### Scanning electron microscopy.

*N. gonorrhoeae* wt, *ΔpilT*, and *pilT*_L201C_ strains were grown in GCB with Kellogg’s supplements on glass coverslips. After 4 h, the medium was removed and the wells were washed gently 3 times with phosphate-buffered saline (PBS). Cells were fixed in PBS containing glutaraldehyde (2%) for 20 min at room temperature. The fixative was then removed, and the coverslip was washed 3 times with PBS. The samples were then processed for scanning electron microscopy as previously described (54).

### Crystal violet retention assays.

Amounts of 5 × 10^8^ bacteria were inoculated into 1 ml GCB plus Kellogg’s supplements in a 12-well dish. After 4, 8, or 24 h, the medium was aspirated and the cultures were washed gently three times with PBS. The cells were fixed in PBS containing 4% methanol-free formaldehyde for 20 min at room temperature. The fixed cells were stained with a 5% crystal violet solution for 20 min at room temperature and then washed gently 5 times with deionized water. Crystal violet retention was measured by washing the stained biofilms with 1 ml methanol and measuring the OD_600_ of the resultant rinse.

### Adhesion and gentamicin protection assays.

ME180 cells (4 × 10^5^ cells/well) were seeded in two 12-well dishes 2 days before the assay. Cells were 100% confluent on the day of infection. Cells were infected at a multiplicity of infection (MOI) of 10 for 4 h. Adhesion and gentamicin protection assays were then performed as previously described (55). At 4 hpi, one plate of cells was used to quantify adhesion frequency, and the second plate was treated with gentamicin (50 µM) for 1 h to quantify gentamicin-resistant (i.e., intracellular) CFU. To determine adhesion frequency, supernatant and cell-associated fractions were serially diluted and plated onto GCB agar plates. CFU were counted 36 h after plating. Adhesion frequency was calculated by dividing the cell-associated CFU by the total input CFU (cell-associated CFU plus supernatant CFU). The cell-associated fraction of the gentamicin-treated samples was plated to quantify gentamicin-resistant CFU. Intracellular frequency was calculated by dividing the gentamicin-protected CFU count by the cell-associated CFU count.

### EGFR phosphorylation assays.

ME180 cells (1 × 10^6^ cells/well) were seeded in 6-well dishes 2 days before the assay. Cells were 100% confluent on the day of infection. Cells were infected at an MOI of 50 for 4 h and then washed with ice-cold PBS and harvested in 200 µl RIPA buffer with protease inhibitors (Roche). For the positive control, 1 ng of epidermal growth factor (Invitrogen) was added to a uninfected well 5 min prior to lysis. Detection of phosphorylated EGFR was performed as previously described (29).

### Real-time PCR analysis.

ME180 cells (1 × 10^6^ cells/well) were seeded in 6-well dishes 2 days before the assay. Cells were 100% confluent on the day of infection. Cells were infected at an MOI of 10 for 2, 4, 6, or 8 h. The medium was then aspirated, and the wells were washed with PBS. One milliliter of Trizol reagent (Invitrogen) was added to each well, and the samples were stored at −20°C until RNA extraction. Total RNA was extracted using the RNeasy kit (Qiagen). Purified RNA samples were eluted in water and stored at −80°C. cDNA was synthesized from 1 µg of total RNA using the iScript Select cDNA synthesis kit (Bio-Rad) according to the manufacturer’s directions. Real-time PCR was performed on an Applied Biosystems Prism 7300 real-time PCR system using TaqMan universal master mix and predesigned TaqMan probes in duplicate. *hbegf, areg*, and *egf* transcript levels were normalized to *gapdh* (encoding glyceraldehyde-3-phosphate dehydrogenase) levels, and expression values were calculated using the comparative cycle threshold (*C*_*T*_) method (Applied Biosystems).

### HB-EGF detection in supernatants.

ME180 cells (1 × 10^6^ cells/well) were seeded in 6-well dishes 2 days before the assay. Cells were 100% confluent on the day of infection. Cells were infected at an MOI of 10 for 2, 4, 6, or 8 h, and total supernatants were collected and stored at −80°C for future analysis. Supernatants were analyzed by dot blot for the presence of soluble HB-EGF using the Bio-Dot microfiltration apparatus (Bio-Rad) fitted with a 0.1-µm nitrocellulose membrane (GE Healthcare Biosciences) rehydrated in TBS. The supernatants were thawed on ice, and 150 µl was added per well for analysis. After the samples were aspirated, the membrane was removed from the apparatus and blocked in TBS containing 5% bovine serum albumin (BSA) for 1 h at room temperature. The membrane was then probed with anti-HB-EGF antibody (R&D Biosystems) in TBS-BSA overnight at 4°C. The secondary antibody (LICOR) was diluted in BSA-TBS and incubated for 1 h at room temperature. The blot was analyzed using densitometry; HB-EGF intensity was normalized to total protein intensity. Recombinant HB-EGF (R&D Biosystems) was serially diluted in PBS to estimate the total HB-EGF concentrations in supernatants by standard curve analysis.

### HB-EGF treatment during infection.

ME180 cells (4 × 10^5^ cells/well) were seeded in 12-well dishes 2 days before the assay. Cells were 100% confluent at the time of infection. Cells were infected at an MOI of 10. At 3.5 hpi, PBS or recombinant HB-EGF (R&D Biosystems) reconstituted in PBS was added to infected cultures (final concentration of 6 ng/ml). Cells were incubated for another 30 min. At 4 hpi, adhesion and gentamicin protection assays were performed as described above.

## SUPPLEMENTAL MATERIAL

Figure S1  Modeling predicts minimal changes in the structure of PilT_L201C_. Download Figure S1, TIF file, 0.2 MB

Figure S2  *N. gonorrhoeae* wt, Δ*pilT*, and *pilT*_L201C_ cells form morphologically distinct microcolonies on human epithelial cells. Images of ME180 cells infected with *N. gonorrhoeae* wt (A), Δ*pilT* (B), or *pilT*_L201C_ (C) at equivalent MOIs were acquired at 4 hpi. Arrowheads indicate locations of microcolonies. Download Figure S2, TIF file, 0.7 MB

Figure S3  *N. gonorrhoeae* Δ*pilT* and *pilT*_L201C_ cells form more robust and morphologically distinct biofilms than the wt. (A) Crystal violet retention assay of wt, Δ*pilT*, and *pilT*_L201C_ biofilms after 4, 8, and 24 h of static growth. Values are the average results from 3 independent experiments. *, *P* < 0.05, Student’s unpaired *t* test. (B) Images of crystal violet-stained wt, Δ*pilT*, and *pilT*_L201C_ biofilms after 24 h of static growth. Download Figure S3, TIF file, 0.4 MB

Figure S4  Map of plasmid used for *pilT* mutagenesis. Download Figure S4, TIF file, 0.1 MB

Table S1  Summary of *N. gonorrhoeae* Tfp retraction-dependent functions. Table S1, DOCX file, 0.01 MB

Table S2  Primers used in this study. Table S2, DOCX file, 0.01 MB

Table S3  Plasmids used in this study. Table S3, DOCX file, 0.01 MB
