# Supplementary figures and images for: Attenuation of the Type IV Pilus Retraction Motor Influences Neisseria gonorrhoeae Social and Infection Behavior
Source: mBio. 2016 Dec 6;7(6):e01994-16. doi: 10.1128/mBio.01994-16 (PMC5142622; doi:10.1128/mBio.01994-16)

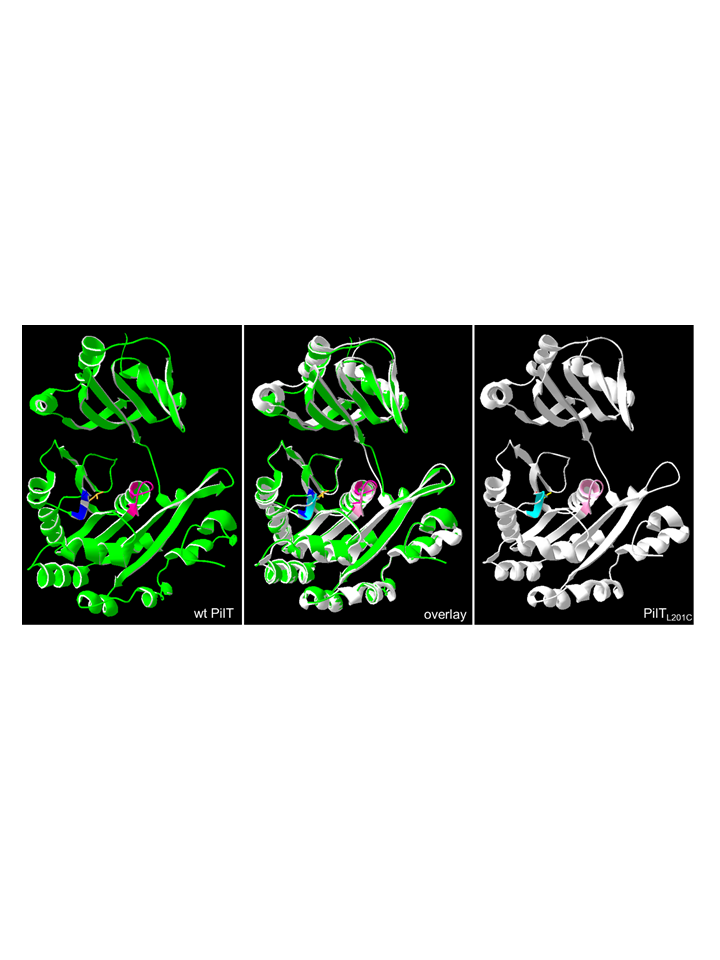

Supplement: Figure S1 — Modeling predicts minimal changes in the structure of PilTL201C. Download [file mbo006163093sf1.tif]

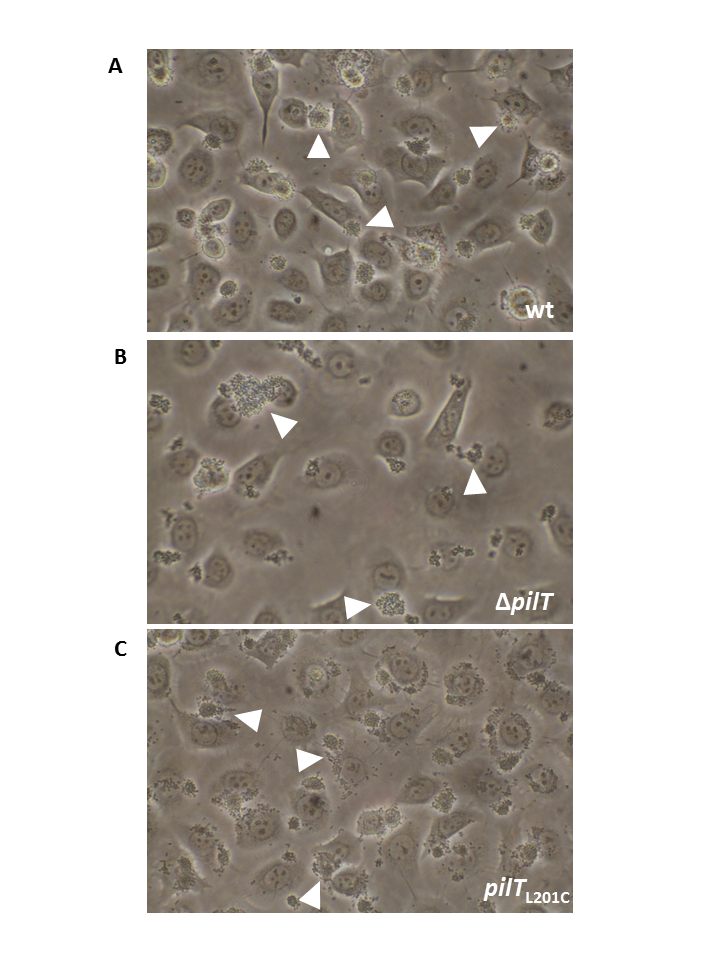

Supplement: Figure S2 — N. gonorrhoeae wt, ΔpilT, and pilTL201C cells form morphologically distinct microcolonies on human epithelial cells. Images of ME180 cells infected with N. gonorrhoeae wt (A), ΔpilT (B), or pilTL201C (C) at equivalent MOIs were acquired at 4 hpi. Arrowheads indicate locations of microcolonies. Download [file mbo006163093sf2.tif]

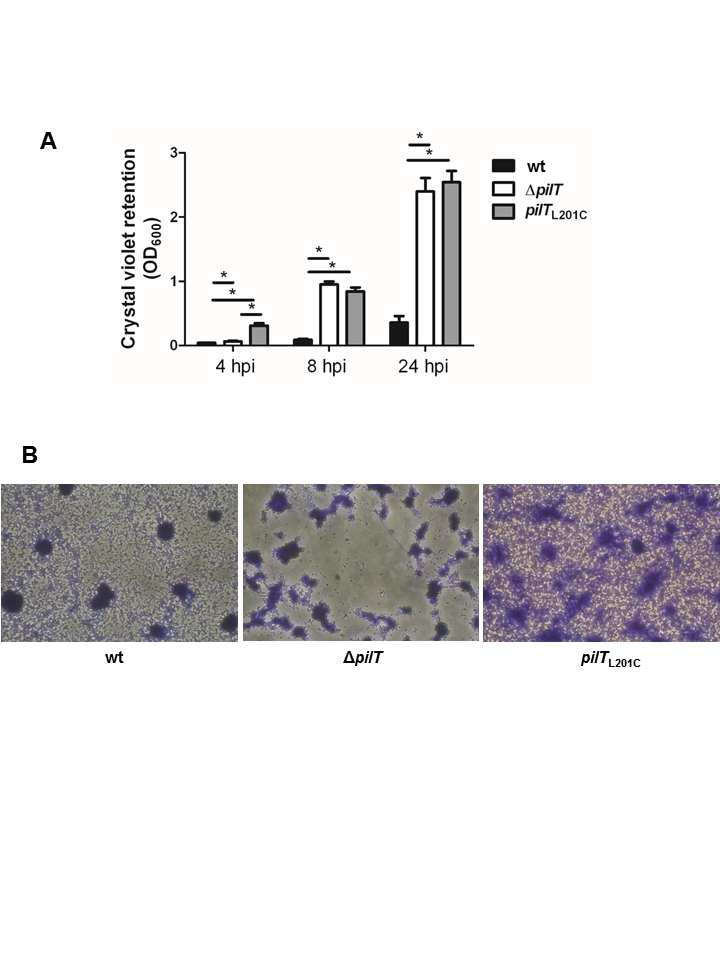

Supplement: Figure S3 — N. gonorrhoeae ΔpilT and pilTL201C cells form more robust and morphologically distinct biofilms than the wt. (A) Crystal violet retention assay of wt, ΔpilT, and pilTL201C biofilms after 4, 8, and 24 h of static growth. Values are the average results from 3 independent experiments. *, P < 0.05, Student’s unpaired t test. (B) Images of crystal violet-stained wt, ΔpilT, and pilTL201C biofilms after 24 h of static growth. Download [file mbo006163093sf3.tif]

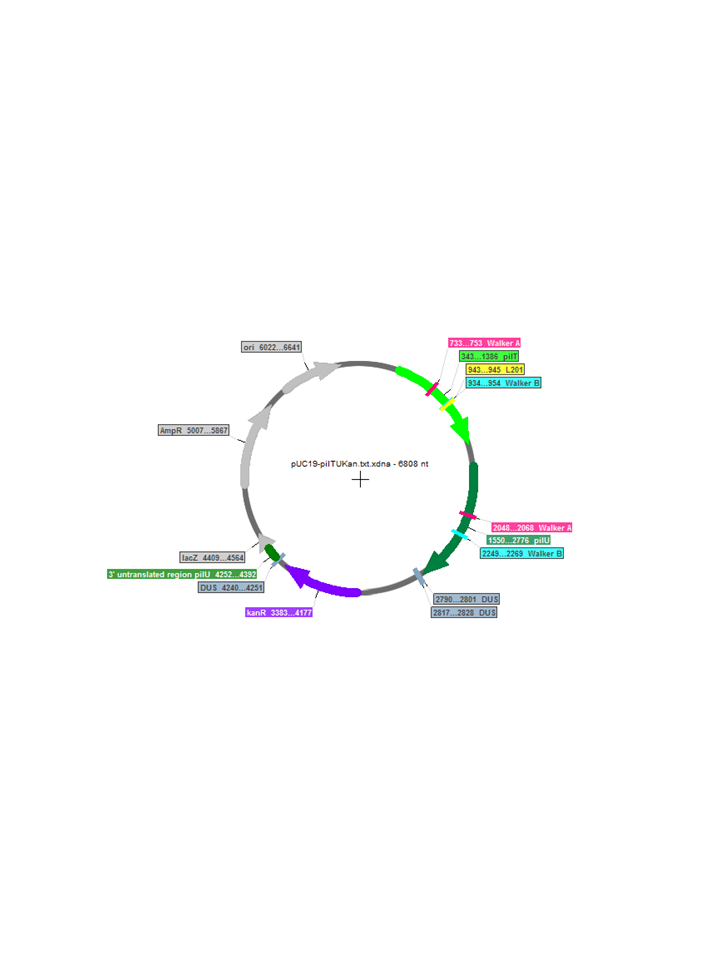

Supplement: Figure S4 — Map of plasmid used for pilT mutagenesis. Download [file mbo006163093sf4.tif]
