# Supplementary material for: Attenuation of the Type IV Pilus Retraction Motor Influences Neisseria gonorrhoeae Social and Infection Behavior
Source: mBio. 2016 Dec 6;7(6):e01994-16. doi: 10.1128/mBio.01994-16 (PMC5142622; doi:10.1128/mBio.01994-16)
Supplement: Table S1 — Summary of N. gonorrhoeae Tfp retraction-dependent functions. [file mbo006163093st1.docx]

**Supplemental Table 1. Summary of Tfp retraction-dependent**

**functions affected in Ngo *pilT*_L201C_.**

| ***Bacterial*** | **wt** | **Δ*pilT*** | ***pilT*_L201C_** |
| --- | --- | --- | --- |
| *pilE* mRNA and protein levels | + | ++ | + |
| Extracellular PilE | + | +++ | ++ |
| Twitching motility | + | - | + |
| DNA uptake | + | - | + |
| Microcolony formation | + | - | -/+ |
| ***Host responses*** | **wt** | **Δ*pilT*** | ***pilT*_L201C_** |
| Intracellular cfu recovered | + | -/+ | -/+ |
| EGFR phosphorylation | + | - | - |
| EGFR recruitment to cortical plaque | + | -/+ | -/+ |
| *hbegf* and *areg* induction | + | - | -/+ |
| HB-EGF release | + | - | - |
