# Supplementary material for: Attenuation of the Type IV Pilus Retraction Motor Influences Neisseria gonorrhoeae Social and Infection Behavior
Source: mBio. 2016 Dec 6;7(6):e01994-16. doi: 10.1128/mBio.01994-16 (PMC5142622; doi:10.1128/mBio.01994-16)
Supplement: Table S2 — Primers used in this study. [file mbo006163093st2.docx]

**Supplemental Table 2. Primers used in this study.**

| **Primer pairs** | **Use** | **Sequence** |  |  |
| --- | --- | --- | --- | --- |
| AH64F | Amplify Ngo *pilT* for cloning into pET28a | agtcaggctagcATGCAGATTACCGACTTACTCGC |  |  |
| AH64R |  | ctgactggatccTTCCTGTTCGGAAGGGTATG |  |  |
| L201CF | Mutagenize *pilT* | GACCCAGACGTTATC**TGC**GTCGGCGAGATGCGC |  |  |
| L201CR |  | GCGCATCTCGCCGACGCAGATAACGTCTGGGTC |  |  |
| AH106F | Amplify Ngo *pilTU* | TCAGGGCGGTATAATCAAGG |  |  |
| AH106R |  | tgatga**ggatcc**GGAAGCGAGGTAATGAGCAG |  |  |
| AH107F | Amplify *kanR* gene | tgatga**ggatcc**GAGTCAGTGAGCGAGGAAGC |  |  |
| AH107R |  | ttcagacggcatGAAATCTCGTGATGGCAGGT |  |  |
| AH108 | Insert *pilU* 3’ region | AGGCCGCCGGCGATGATGCCGAGTACGAAGGGCATGGCGAGCTTGGGTTCGCCTAGCTGCCAGACGATGGAGGCGGCGGTAAAGACACTGGCGAAAACGGttcagacggcatGAAATCTCGTGATGGCAGGT |  |  |
| MR250 | Detect *16S* transcript | GAGTGTGTCAGAGGGAGGTG | |  |
| MR251 |  | TTTAGGGCGTGGACTACCAG |  |  |
| MR258 | Detect *pilT* transcript | CTTCGGCGCTAAAAACAAAG | |  |
| MR259 |  | TAGATTTTCCGCTGGTGGTC |  |  |
| AH48F | Detect *pilU* transcript | CAACCAAATACTGATTGACATCG |  |  |
| AH48R |  | TACAGTTCGAAAAGGTTTTGGTC |  |  |
| MR260 | Detect *pilT2* transcript | ATGGCGGCGTTGAAAAATAC | |  |
| MR261 |  | GAGGCGTTGCGAAATAAAC |  |  |
| AH42F | Detect *pilE* transcript | CGTCACCGAGTATTACCTGAATC |  |  |
| AH42R |  | GCCGTTTTTAACTTCAACCTCTT |  |  |
| AH41F | Detect *pilF* transcript | GTTTAATATTGCCAGTTCGGTCA |  |  |
| AH41R |  | CAATCTTTTGCAAGATCCTCATC |  |  |
