# Supplementary material for: Attenuation of the Type IV Pilus Retraction Motor Influences Neisseria gonorrhoeae Social and Infection Behavior
Source: mBio. 2016 Dec 6;7(6):e01994-16. doi: 10.1128/mBio.01994-16 (PMC5142622; doi:10.1128/mBio.01994-16)
Supplement: Table S3 — Plasmids used in this study. [file mbo006163093st3.docx]

**Supplemental Table 3. Plasmids used in this study.**

| **Plasmid** | **Use** |
| --- | --- |
| pET28a-*pilT* | Over-expression of His_6_-PilT |
| pET28a-*pilT*_L201C_ | Over-expression of His_6_-PilT_L201C_ |
| pUC19-*pilTU*-*kan* | Vector to insert *pilT*_L201C_ into Ngo |
